# Enterocele Presenting as Anterior Rectal Prolapse: Resolution with Vaginal Repair

**DOI:** 10.1155/2020/1796365

**Published:** 2020-01-02

**Authors:** Geoffrey D. Towers, Candace Benoit, Rose Maxwell, Jerome Yaklic

**Affiliations:** Department of Obstetrics and Gynecology, Wright State University Boonshoft School of Medicine, 128 E. Apple Street, Suite 3800 CHE, Dayton, OH 45409, USA

## Abstract

An enterocele is a pelvic hernia formed from the separation of endopelvic fascia, associated with the posterior or anterior vaginal fornix, and most commonly located in the posterior superior vaginal segment. Rectal prolapse is a debilitating condition in which the mucosa of the rectum protrudes circumferentially from the anus. Surgical repair is the recommended treatment for rectal prolapse, and though there are many different surgical options, there is no consensus on which approach is best. We present a case of anterior rectal prolapse due to enterocele which was treated by correction of enterocele with a vaginal approach and propose some clinical features and diagnostic techniques that may distinguish this entity from traditional rectal prolapse.

## 1. Introduction

An enterocele is a pelvic hernia formed from the separation of endopelvic fascia, associated with the posterior or anterior vaginal fornix, and most commonly located in the posterior superior vaginal segment [1]. As a true hernia, an enterocele may contain abdominal contents and is often found concurrently with other pelvic floor defects. It is estimated that up to 3% of females in the United States suffer from some type of vaginal prolapse [2], usually presenting with complaints of a vaginal bulge or pressure. In contrast, rectal prolapse occurs when anal mucosal or rectal tissue protrudes circumferentially through the anal orifice and is typically a continuum starting with internal intussusception and progressing to eventual full-thickness procedentia. This complete concentric prolapse is uncommon, present in less than 0.5% of the population [3], and occurs more frequently in females and the elderly. Symptoms are variable and can include physical bulge, defecatory dysfunction, stool abnormalities, pain, and bleeding [4]. It is common for patients with rectal prolapse to have other coexisting pelvic floor disorders. Quality of life is universally negatively impacted [5]. Rectal prolapse is diagnosed clinically based on history and physical examination findings, with surgical repair as the treatment of choice [4]. A myriad of surgical procedures is available to treat rectal prolapse, with open abdominal, perineal, and endoscopic approaches both with and without graft augmentation [4]. Although a vaginal approach to rectal prolapse repair is not typically described, a single case was reported in 2017 by Devakumar et al. in which successful repair of anterior rectal prolapse was accomplished with a vaginal approach, including enterocele repair with Moschowitz culdoplasty [6]. We present a similar case of anterior rectal prolapse also surgically corrected with vaginal reconstructive surgery. Written informed consent for publication of this case was obtained from our patient.

## 2. Case Presentation

**Figure 1:**
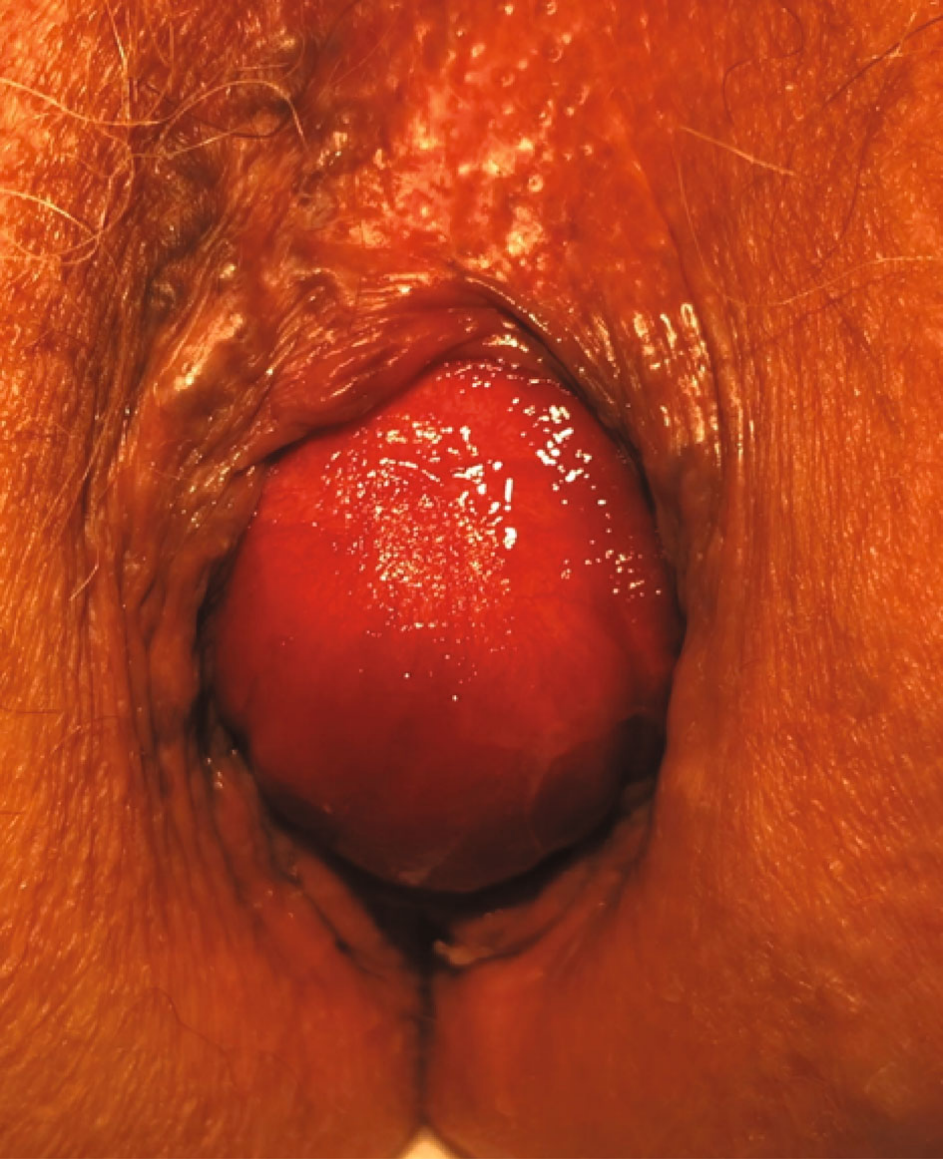
84-year-old female with isolated anterior rectal prolapse due to enterocele. Note the smooth surface with lack of concentric folds.

An 84-year-old postmenopausal white female (gravida 3, para 2) was referred to our urogynecology clinic for evaluation and treatment of a persistent rectal bulge (Figure 1) ([Supplementary-material supplementary-material-1]). She had noticed this bulge for over 10 years and initially thought that it was a hemorrhoid. She had been evaluated by a colorectal surgeon 10 years prior to presentation who recommended an abdominal resection rectopexy. The patient elected to not pursue surgery at that time. She described a bulge at the anus with the size of a lemon which worsened with physical activity and reported accidental leakage of bowel contents consisting of pellet-formed stool. She was sometimes unable to differentiate between the bulge and stool. She reported significant adverse impact on her quality of life, including impairment of her ability to mow her lawn or to exercise. Additionally, she reported anal laxity, constipation, and occasional bright red blood per rectum. She denied any urinary symptoms or vaginal prolapse.

Pelvic exam revealed an atrophic vagina with no significant anterior or posterior vaginal wall prolapse. Her cervix was absent surgically, and her pelvic organ prolapse quantification was as follows: GH 2, PB 2, Aa -3, Ba -3, Ap -3, Bp -3, C -7, D n/a, and TVL 7. Rectal exam showed normal perianal skin and sphincter tone. With Valsalva, she demonstrated significant anterior rectal wall prolapse with palpable contents consistent with small bowel enterocele in the rectovaginal space. Prolapse was approximately 6 cm in size with the anterior rectal wall extending outside the anal verge and no evidence of posterior or circumferential rectal prolapse. Endoanal ultrasound showed normal levators, intact external anal sphincter circumferentially, and attenuation of the internal anal sphincter. There were no discernible masses, but the patient was unable to produce her rectal prolapse with the ultrasound transducer in place. Transperineal ultrasound was used to dynamically evaluate the perineal and rectal anatomy and demonstrated a large enterocele prolapsing between the vagina and the rectum with apparent fat or small bowel contents. Prolapse at the anterior rectal wall was directly associated with this enterocele. Defecography and dynamic MRI were not available at our facility.

The patient was evaluated by a colorectal surgeon for opinion on treatment plans. This surgeon indicated that a rectopexy would be acceptable, but it would be reasonable to attempt to repair the enterocele first to see if her symptoms would resolve and to potentially spare the patient from a more morbid procedure. The patient indicated a desire to proceed with an attempt at repair with the vaginal approach.

**Figure 2:**
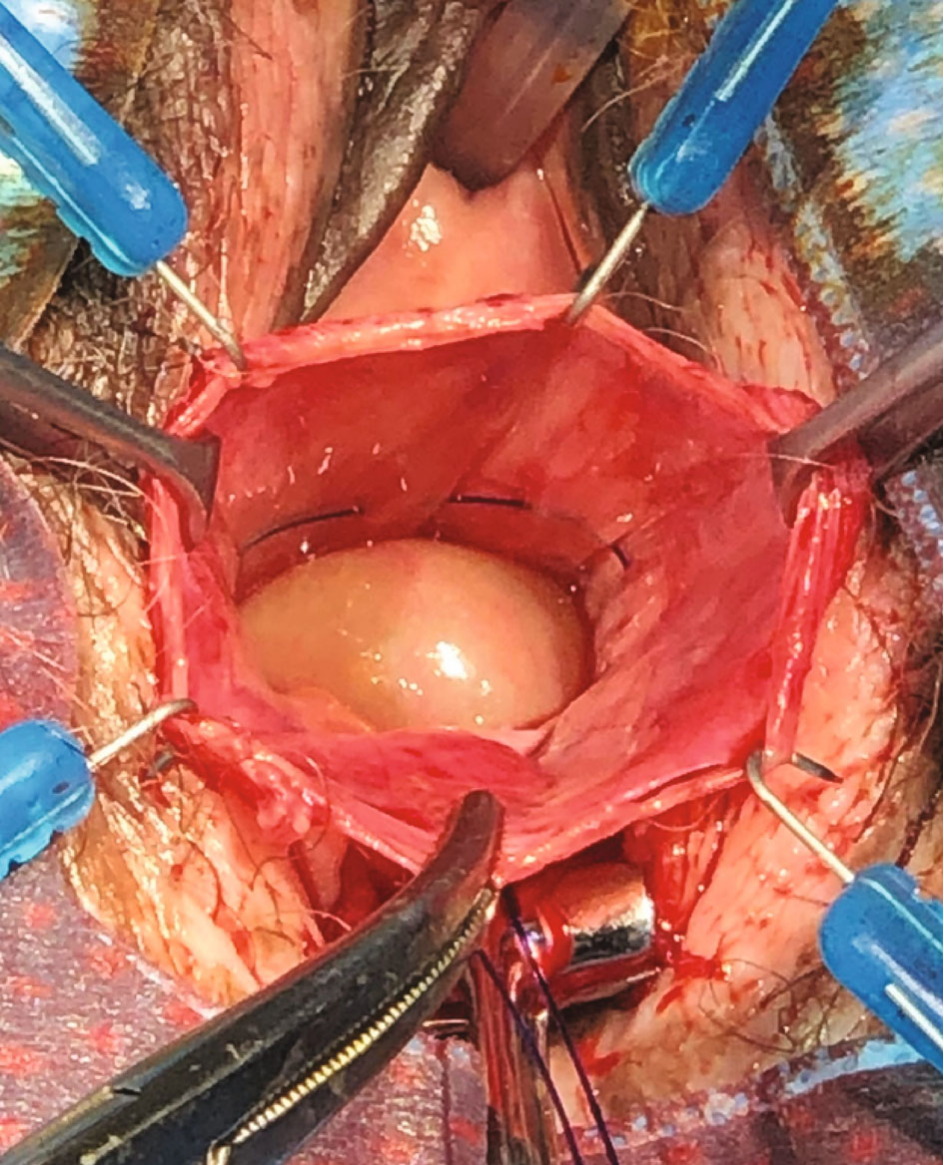
Purse-string repair of enterocele using delayed absorbable monofilament suture.

**Figure 3:**
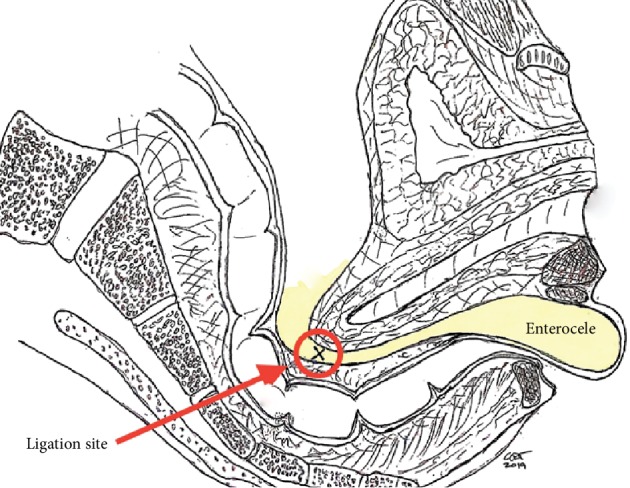
Location of the repair site for rectal enterocele.

**Figure 4:**
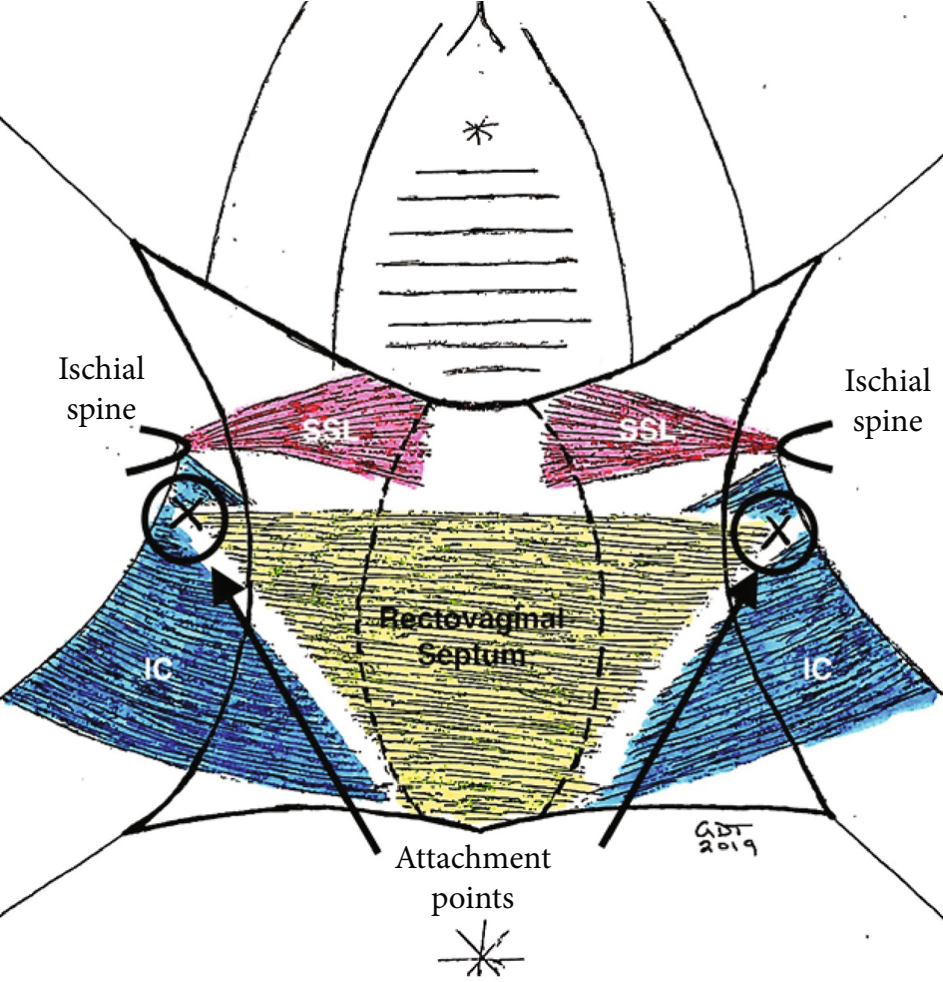
Fixation location for iliococcygeus suspension sutures. A finger is placed on the ischial spine and a suture capturing device used to place a delayed absorbable or permanent suture into the iliococcygeus tendon at the marked location. IC = iliococcygeus muscle; SSL = sacrospinous ligament.

**Figure 5:**
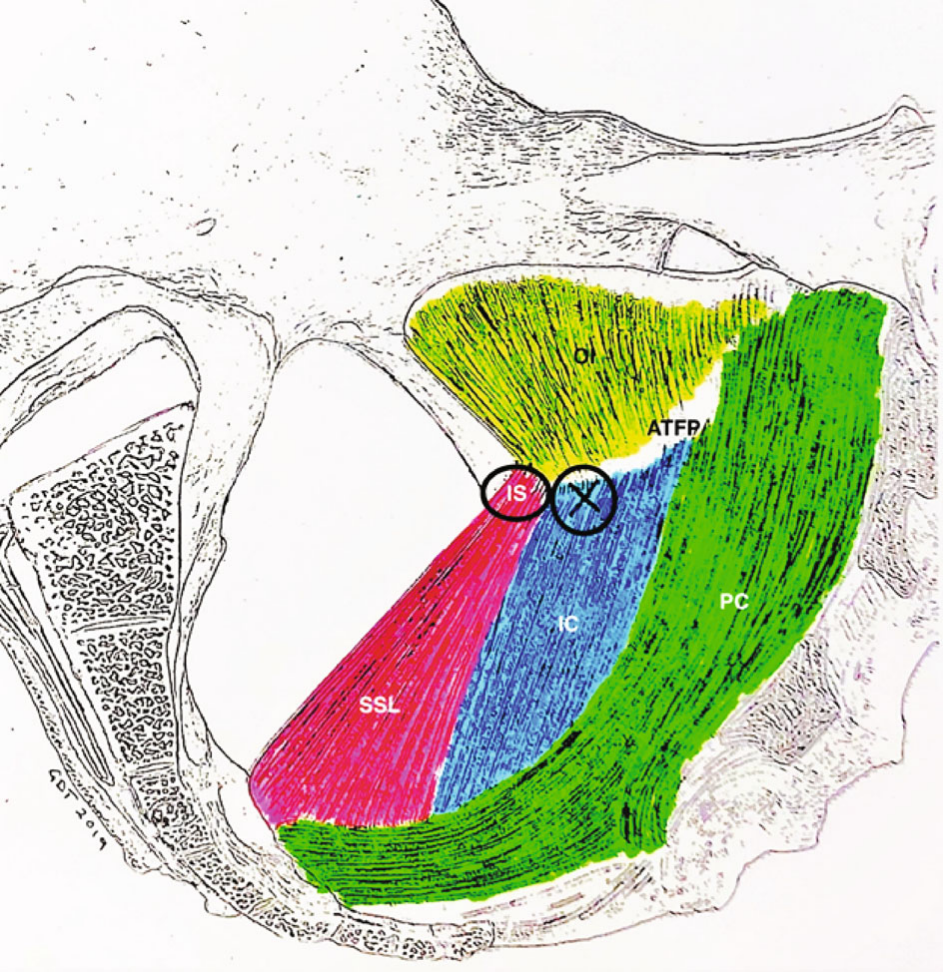
Location of placement of fixation suture for iliococcygeus suspension. Suspension in this case was necessary due to inferior/posterior prolapse of the rectovaginal septum associated with the rectal enterocele. IS = ischial spine; PC = pubococcygeus; IC = iliococcygeus; SSL = sacrospinous ligament; ATFP = arcus tendineus fasciae pelvis; OI = obturator internus.

The patient was admitted to the hospital and anesthetized for surgery. A vertical posterior vaginal incision was made, extending into the perineal body. The vaginal epithelium was separated from the underlying endopelvic fascia, and dissection was performed superiorly and laterally to the level of the ischial spines and levator ani. A large enterocele was identified, opened, ligated with two purse-string sutures of delayed absorbable monofilament suture, and then excised (Figures 2 and 3). A suture capturing device was used to pass a polypropylene suture through the levator tendon immediately distal to the ischial spine bilaterally, following which these sutures were attached superiorly and laterally to the rectovaginal septum completing the iliococcygeus suspension in a tension-free manner (Figures 4 and 5). Small site-specific rectovaginal septal defects were repaired. The vaginal epithelium was then closed, and the perineal body was reconstructed.

Postoperatively, the patient recovered well and was discharged home on the first postoperative day with complete resolution of her rectal prolapse symptoms. She was seen at 1 month and 3 months postoperatively and by 3 months had resumed her normal activities of daily living with complete symptom resolution and normal defecation without incontinence.

## 3. Discussion

Rectal prolapse is a challenging and debilitating disease, with significant negative impact on sufferers. A significant number of surgical procedures (>100) are available to apply in these cases, and there is no consensus on which procedure is best [7]. Current practice is to tailor the treatment to the individual patient, accounting for medical comorbidities and patient desires with careful surgical planning [4], and to involve experts in the pelvic floor for surgical reconstruction should other pelvic floor defects be identified [7]. Most rectal prolapse literature, however, addresses full-thickness rectal prolapse specifically, and there is very little which addresses isolated anterior rectal prolapse. Indeed, ours is a case substantially like that reported by Devakumar et al. in 2017, where isolated anterior rectal prolapse was treated with repair of enterocele using a vaginal approach [6], and is only the second such case reported in the literature. In their case, treatment of the enterocele was with a Moschowitz culdoplasty and mesh vaginal suspension, while we were able to close the defect with purse-string ligation of the enterocele and suture suspension of the posterior vaginal wall. It is possible that any of the described enterocele repair techniques would be effective in a similar setting, depending on the individual patient.

As noted in Devakumar's discussion, Moschowitz proposed in 1912 that rectal prolapse could be the result of a deep pouch of Douglas which would allow pressure on the anterior rectal wall to cause the rectum to evert outside the anus [6, 8], and certainly this mechanism appears to be applicable in these two cases. The question remains, however, whether rectal prolapse initiated by this mechanism would progress to full-thickness circumferential prolapse or whether isolated anterior rectal prolapse in the setting of enterocele is a separately identifiable condition that justifies a different surgical approach.

**Figure 6:**
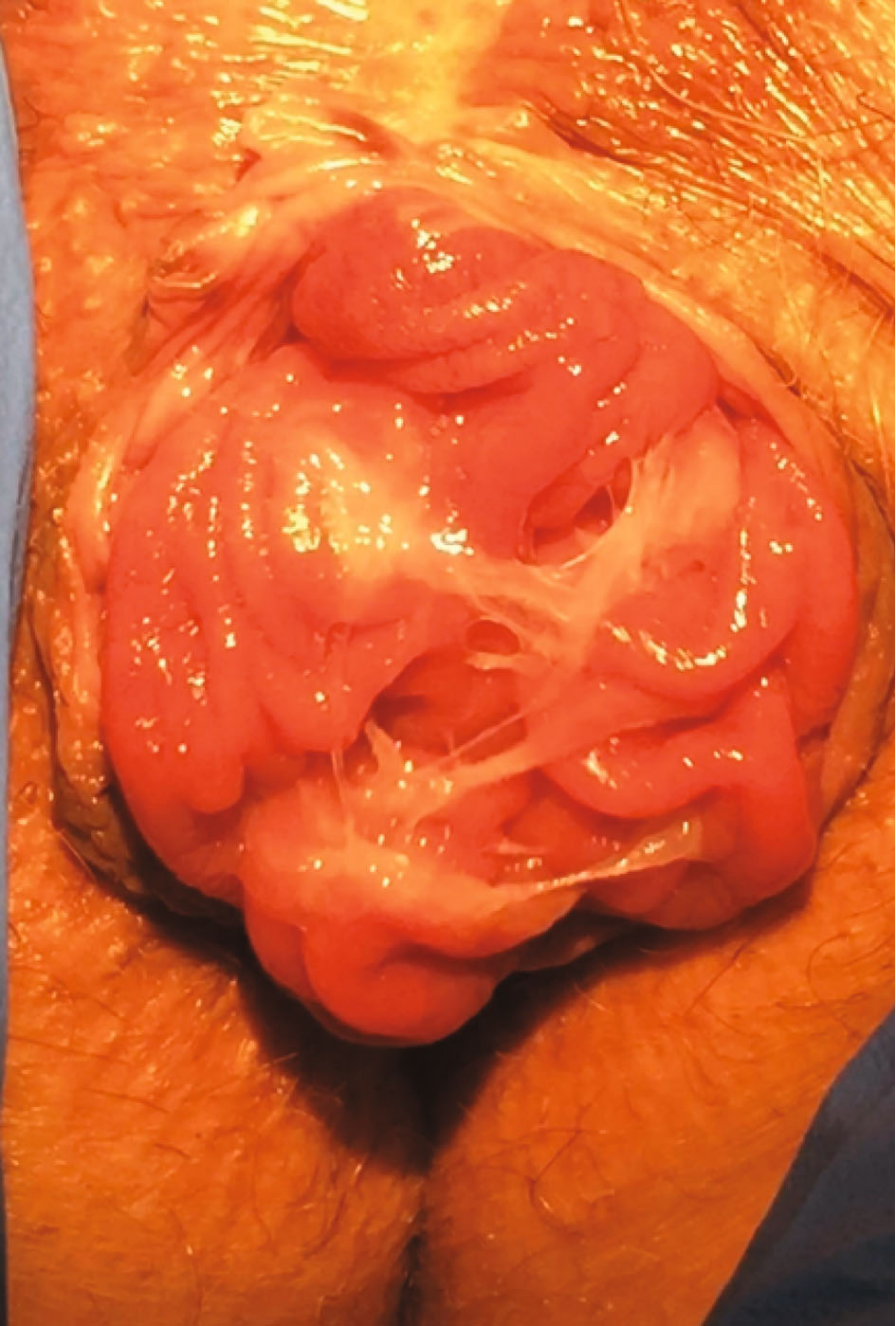
A different patient with full-thickness rectal prolapse due to intussusception. Note the concentric mucosal folds.

It is important to note that there are likely significant differences between these two cases and traditional circumferential rectal prolapse. It is possible that these are unique clinical entities. In looking at the images in Devakumar's case and in our case (Figure 1) ([Supplementary-material supplementary-material-1]), it can be easily seen that rectal presentation of an enterocele differs clinically from full-thickness rectal prolapse that results from intussusception (Figure 6) ([Supplementary-material supplementary-material-1]). Note particularly that the concentric rings seen in traditional rectal prolapse are absent in these cases, suggesting lack of full-thickness prolapse and a difference in the mechanism (herniation of enterocele rather than intussusception). In our case, bowel contents of the enterocele could be easily palpated, and the diagnosis was supported by transperineal ultrasound imaging. Though defecography and dynamic MRI were not available to us and though such imaging could be useful if the diagnosis were in doubt, we did not feel they were necessary in this case.

## 4. Conclusion

Enterocele presenting as anterior rectal prolapse appears to be a rare condition, but one that may be amenable to a vaginal approach to treatment rather than traditional rectal prolapse repair. Careful physical examination is crucial. As a key physical exam finding, absence of circumferential mucosal folds and isolated anterior prolapse may herald the presence of an enterocele and should prompt an appropriate evaluation. There are no studies or case series describing the ideal approach for surgical correction of this rare presentation, and as in the case of any rectal prolapse, careful individualized evaluation and planning are warranted. Further research into the mechanism of rectal prolapse and the role of enterocele is needed. In this case, a vaginal surgical approach resulted in excellent results, at least in the short term, and allowed the avoidance of potentially more invasive and morbid procedures.

## Supplementary Materials

Supplementary 1 Video Clip 1: subject patient with enterocele causing anterior rectal prolapse.

Supplementary 2 Video Clip 2: comparison patient with full-thickness circumferential rectal prolapse.
